# Follow-up of non-palpable testicular incidentalomas under 1 cm: does growth rate differentiate malignant and non-malignant lesions?

**DOI:** 10.1007/s00330-024-10981-4

**Published:** 2024-07-30

**Authors:** Michele Bertolotto, Irene Campo, Simon Freeman, Francesco Lotti, Dean Y. Huang, Laurence Rocher, Lucio Dell’Atti, Massimo Valentino, Pietro Pavlica, Paul S. Sidhu, Lorenzo E. Derchi

**Affiliations:** 1 Department of Radiology, University of Trieste, Ospedale Di Cattinara, Strada di Fiume 447, 34149 Trieste, Italy; 2 University Hospitals Plymouth NHS Trust, Imaging Directorate, Derriford Hospital, Derriford Road Crownhill, Plymouth, Devon PL6 8DH UK; 3 Department of Experimental and Clinical Biomedical Sciences “Mario Serio”, University of Florence, Largo Brambilla, 3, 50134 Florence, Italy; 4 Andrology, Women’s Endocrinology and Gender Incongruence Unit, Center for Prevention, Diagnosis and Treatment of Infertility, Careggi University Hospital, 50139 Florence, Italy; 5 Department of Imaging Sciences, School of Biomedical Engineering and Imaging Sciences, Faculty of Life Sciences and Medicine, Department of Radiology, King’s College London, King’s College Hospital, London, UK; 6 Service de Radiologie, APHP, Hôpital Antoine Béclère, BIOMAPS, Université Paris-Saclay, 157 rue de la porte de trivaux, 92140 Clamart, France; 7 Unit of Quality and Risk Management, Department of Urology, University-Hospital of Marche, Street Conca 71, 60126 Ancona, Italy; 8 Department of Radiology, Ospedale Sant’Antonio Abate, Via Giobatta Morgagni 18, 33028 Tolmezzo, UD Italy; 9 Private Hospital S. Maria Maddalena, Via Gorizia 2 – S. Maria Maddalena, 45030 Occhiobello, RO Italy; 10 Dipartimento di scienze della salute – DISSAL, Università di Genova, Via Pastore 1, 16132 Genova, Italy

**Keywords:** Testicular incidentaloma (imaging), Non-palpable testicular tumours (imaging), Testis lesion (growth rate), Incidental testicular tumour (histology)

## Abstract

**Objective:**

To determine whether small, incidentally detected testicular lesions can be safely followed up, by assessing growth rate and volume threshold for benign vs. malignant lesions.

**Methods:**

This retrospective observational study includes a consecutive series of 130 testicular incidentalomas < 1 cm and with negative tumour markers identified from October 2001 to November 2022, which were initially followed up with ultrasound. A total of 39 cases proceeded to surgery during the study period, either due to lesion growth (*n* = 28) or patient preference/recommendation by the referring urologist (*n* = 11). For the lesions that were growing, specific growth rate (SGR) and doubling time (DT) were calculated assuming an exponential growth pattern. In addition, the velocity of increase of the average diameter (∆D_av_) and of the maximum diameter (∆D_max_) were calculated.

**Results:**

Of the 130 nodules that were initially followed up, six disappeared, eight were reduced in size, eighty-eight were stable, and twenty-eight increased in size. For operated nodules all 18 malignant tumours, 8/9 benign tumours, and 2/12 surgically proved non-neoplastic lesions were growing. The best cut-off values of the growth indicators to differentiate between malignant and non-malignant histology were 3.47 × 10^−3^%volume/day, ≤ 179 days, > 10 × 10^−3 ^mm/day, and > 5 × 10^−3 ^mm/day for SGR, DT, ∆D_max_, ∆D_av_, respectively.

**Conclusions:**

Malignant and non-malignant small incidentalomas can be effectively differentiated based on growing parameters, even though overlap exists. An increase of the maximum diameter of about 1 mm and 2 mm in three months and in six months, respectively, suggests malignancy.

**Clinical relevance statement:**

Growing parameters allow an educated assessment of benign and malignant small testicular incidentalomas. Non-aggressive management is justified and safe when follow-up includes self-examination and tumour marker assessment to reduce the risk of interval tumour growth.

**Key Points:**

- *Small, non-palpable and asymptomatic testicular nodules <* *1* *cm are unexpectedly discovered during scrotal ultrasound*.
- *Growth indicators estimate the potential malignancy, even though overlap with non-malignant lesions exists*.
- *Non-growing incidentalomas can be safely followed up*.

## Introduction

Small (< 1 cm), asymptomatic testicular lesions are often discovered incidentally during a scrotal US investigation performed for another purpose. Only about 30% of these incidentalomas are malignant [1]. The smaller the lesion, the lower is the chance of it being malignant. Virtually all lesions < 3 mm are benign, while lesions < 5 mm are benign in 87% of cases [1].

According to the current EAU Guidelines on Testicular Cancer, testis-sparing surgery (TSS) together with frozen section examination can be offered to patients with a high likelihood of having a benign testicular tumour, but orchidectomy is still considered the treatment of choice in patients with solid testicular nodules, and lesion follow-up is not recommended [2]. However, urological practice is evolving towards a less aggressive approach that incorporates serial US monitoring [3, 4].

Since very small lesions may actually be malignant tumours, the purpose of this less aggressive approach is to differentiate lesions that can be safely followed up from those that require surgery, either TSS or orchidectomy. The growth rate of a lesion is used to indicate potential malignancy, whether or not it is associated with positive tumour markers [4]. Testicular tumours are usually considered to be fast-growing lesions whereas benign tumours and/or non-neoplastic lesions grow slowly, remain stable, reduce in size, or even disappear during follow-up. This is commonly observed in clinical practice; however, there is no robust supporting data in the literature, and the reported fast doubling times (ranging from 10 to 30 days) of testicular neoplasms relate to the growth characteristics of metastatic lymph nodes, rather than the primary tumour [5].

Since the growth rate of small testicular tumours is unknown, no thresholds have been established to distinguish between benign and malignant lesions. As a consequence, differentiation based on the rate of growth is invalidated.

In the clinical practice of the hospitals involved in this study, testis-sparing surgery is recommended for incidentally detected non-palpable lesions ≥ 1 cm and for lesions < 1 cm that are growing [6]. By convention, an overall increase in the greatest diameter ≥ 1 mm during two consecutive follow-up investigations is considered unequivocal growth.

In this study, a series of small, incidentally detected non-palpable testicular lesions with negative tumour markers was retrospectively investigated. The lesions were subject to longitudinal US follow-up. In cases where surgical resection was undertaken during the study period the final histology was recorded. The aim was threefold: (a) to substantiate whether small, incidentally detected testicular lesions can be safely followed up; (b) to assess the growth rate of surgically proven benign, malignant, and non-neoplastic lesions; (c) to investigate whether a threshold could be identified for reliable differentiation between benign/non-neoplastic lesions and malignant tumours based on growth characteristics.

## Materials and methods

This retrospective observational study was approved by the Ethics Committee of the University of Trieste (verb128, 27/02/2023). Due to the retrospective nature of the study, patient informed consent was waived. Data were processed without any patient-identifying information.

From October 2001 to November 2022, in seven diagnostic centres, patients with incidentally detected, non-palpable testicular lesions measuring < 1 cm in maximum diameter and with negative tumour markers, who were initially scheduled for active surveillance with US, were included in the study. Only lesions with ‘solid’ components were included, simple cysts were excluded. Three patients lost to follow-up were also excluded. In the clinical practice of the hospitals involved in this study, other imaging techniques are not routinely used for the follow-up of these nodules.

### Data analysis

US images obtained during the follow-up studies were retrieved for retrospective review. Patients’ age at the time of the first examination and histologic results of the operated lesions were also retrieved from the archives. A retrospective review of US images was performed on a per-patient basis by a radiologist who was unaware of the histology of the lesion. If the patient had multiple testicular lesions, the largest was considered. The reviewer was asked to measure the lesions during follow-up and to assess whether they were stable, shrinking, growing, or disappearing. Lesions were considered stable if there was no change or a decrease of < 1 mm during two consecutive follow-up investigations, as shrinking when their maximum diameter decreased by > 1 mm, or as growing if the greatest diameter increased in the first follow-up examination, and then increased again in a second follow-up examination with an overall increase of 1 mm or more.

For the lesions that were growing, the three diameters of the target nodules were measured, and volume was calculated by applying the ellipsoid formula. In cases where two orthogonal images were not available for measurement of the three diameters, the two minor axes were considered equal, and the oblate ellipsoid formula was applied to calculate the volume. The specific growth rate (SGR) and doubling time (DT) were calculated as previously described, assuming an exponential growth of the lesion [7]. SGR (%volume/day) is defined as the percentage volume increase per unit of time. DT (in days) is defined as the amount of time taken for the lesion to double in size. Since the uncertainty of volume estimation can be high for millimetric nodules [8], the velocity of increase of the average diameter (∆D_av_) and of the maximum diameter (∆D_max_) of the nodules were also calculated. The formulas used for measuring these parameters are reported in Table 1.

**Table 1:** Definition of the parameters used for quantification of lesion growth rate

Parameter	Equation	Definition
Specific growth rate (SGR)	ln(V2/V1)/(t2-t1) (%volume/day)	Percentage volume increase per unit of time
Doubling time (DT)	ln(2)/SGR (Days)	Amount of time it takes for the lesion to double in volume
Velocity of increase of the maximum diameter (∆D_max_)	(D_max2_-D_max1_)/(t2-t1) (mm/days)	Velocity of increase of the maximum diameter
Velocity of increase of the average diameter (∆D_av_)	(D_av2_-D_av1_)/(t2-t1) (mm/days)	Velocity of increase of the average diameter

V1, D_max1_, D_av1_: lesion volume, maximum diameter and average diameter at time t1

V2, D_max2_, D_av2_: lesion volume, maximum diameter and average diameter at time t2

### Reference procedure

The lesions which reduced in size or were stable during a follow-up of 1 year or more were considered presumably benign. Histology was available in 39 patients (28 growing and 11 stable lesions).

### Statistical analysis

Statistical analyses were performed using MedCalc for Windows, version 19.3.1 (MedCalc Software). Student’s *t*-test, and receiver operating characteristic (ROC) curve analysis with Youden’s J statistics were used.

Size differences of malignant and non-malignant nodules when first identified at ultrasonography, and differences among the growth indicators were assessed using the Student’s *t*-test. The sensitivity, specificity, and predictive values to assess malignancy for different growth indicators were tested using a ROC curve analysis. The Youden’s index was applied to identify the cut-off values that maximise both sensitivity and specificity.

## Results

A total number of 130 consecutive patients (median age, 38 years; range, 16–78 years) with incidentally detected, non-palpable testicular lesions < 1 cm of maximum diameter were initially scheduled for active surveillance. In 37/130 (28.5%) patients who had multiple small lesions, the largest was considered for the purpose of this study. The remaining 93 patients had a single lesion. In most cases (101/130, 78%) follow-up was carried out following European Society of Urogenital Radiology (ESUR) recommendations [9], and patients were monitored every three months for 12 months and then annually. In the remaining 29 patients, one or more examinations were missed during follow-up. Surgery was scheduled during follow-up for 39/130 (30.0%) lesions because the lesion was growing (*n* = 28), or because of patient preference or recommendation of the referring urologist (*n* = 11).

**Fig. 1:**
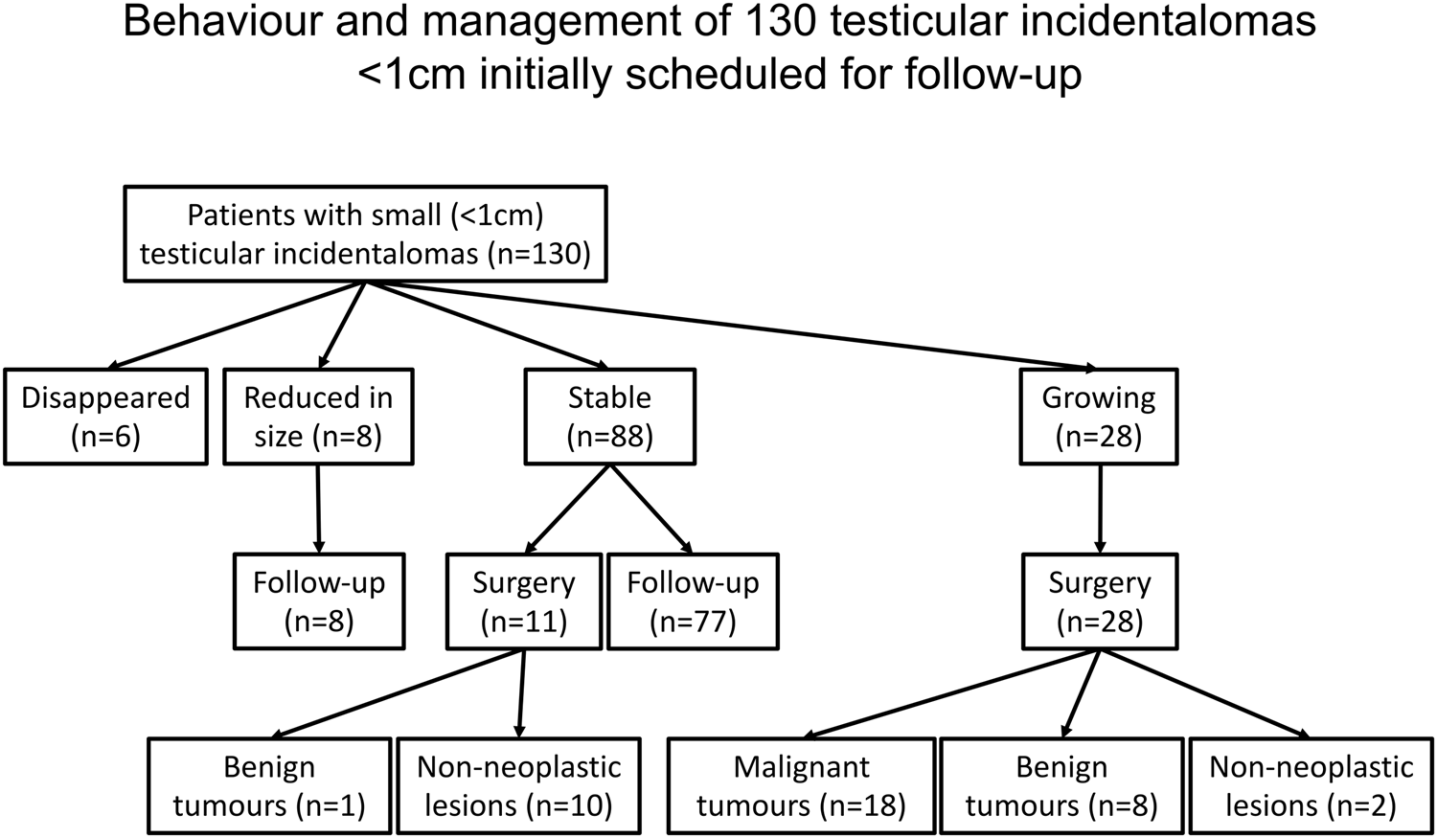
History of 130 testicular incidentalomas < 1 cm. During the follow-up six lesions disappeared, eight reduced in size, 88 were stable, and 28 increased in size. Thirty-nine lesions were operated, 11 were stable, and 28 growing. The 11 stable lesions were one benign leydigoma and ten non-neoplastic lesions. Eighteen growing lesions were malignant tumours, eight were benign tumours, and two were non-neoplastic lesions

**Fig. 2:**
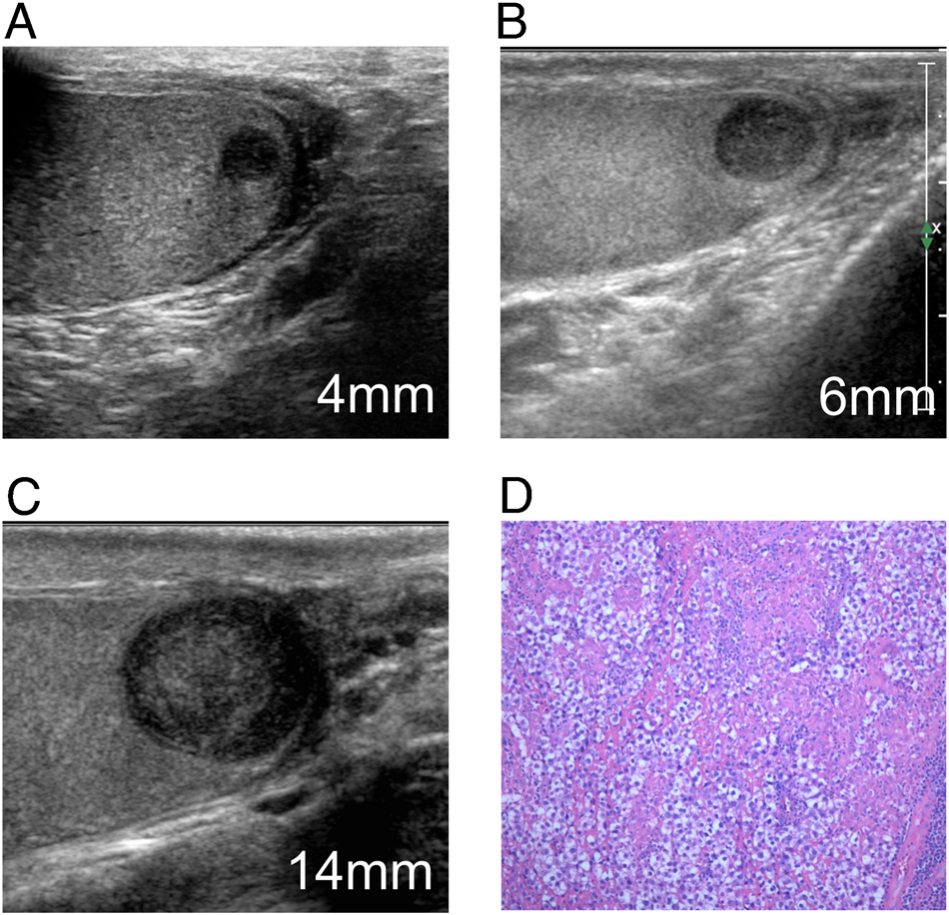
Non-palpable, incidentally detected testicular lesion which displayed rapid growth during the follow-up. In the first examination (**A**) the lesion had a maximum diameter of 4 mm, while the diameter was 6 mm and 14 mm after 3 months (**B**) and 6 months (**C**). A seminoma was found at surgery (**D**)

**Fig. 3:**
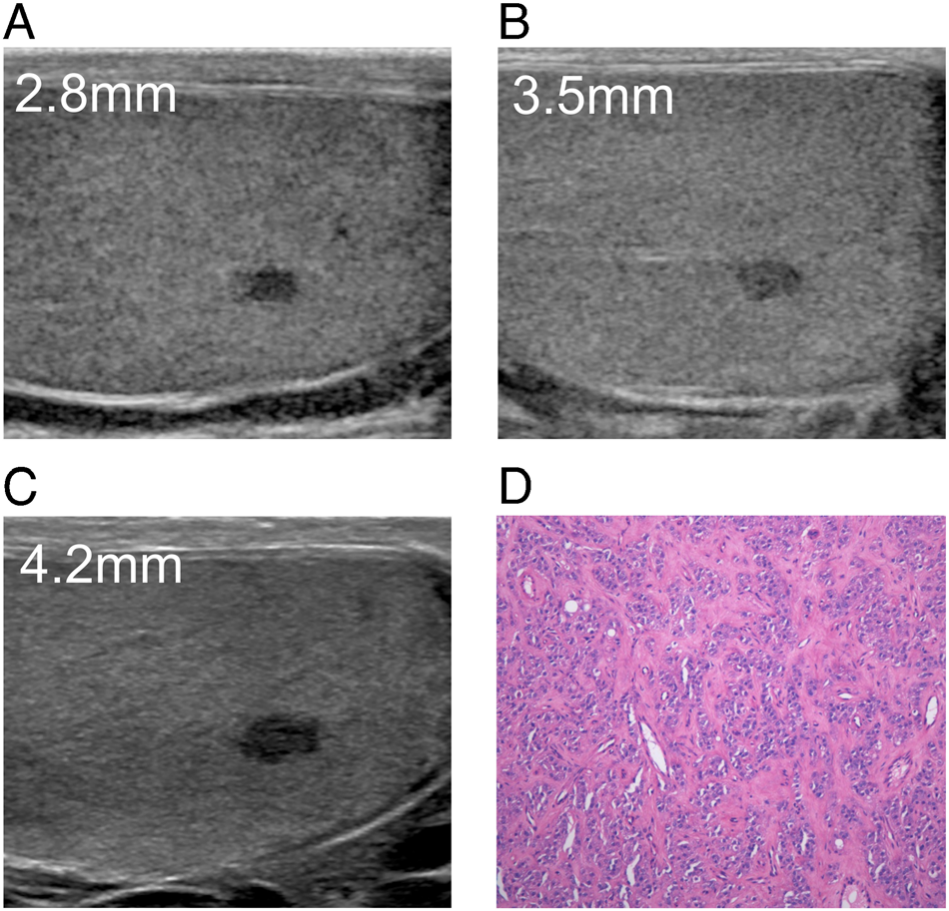
Non-palpable, incidentally detected testicular lesion which displayed a slow growth during the follow-up. In the first examination (**A**) the lesion had a maximum diameter of 2.8 mm, while the diameter was 3.5 mm and 4.2 mm after 3 months (**B**) and 6 months (**C**) during the follow-up. A benign leydigoma was found at surgery (**D**)

Figure 1 summarises the behaviour of the 130 incidentalomas, their management, and histology of the 39 operated lesions. Of the 130 testicular lesions which were initially followed up, 6 disappeared, 8/130 (6.2%) reduced in size, 88/130 (67.7%) were stable, and 28/130 (21.5%) increased in size. (Figs. 2–3). The duration of follow-up in non-operated lesions ranged from 1 to 22 years. Table 2 details the lesions with histology. The stable operated lesions were classified as Leydig hyperplasia (*n* = 6), fibrosis (*n* = 3), granulomatous orchitis (*n* = 1), and benign leydigoma (*n* = 1). Of the 28 growing lesions, 18/28 (64.3%) were malignant tumours (seventeen seminomas, and one mixed germ cell tumour) (Fig. 2), 8/28 (28.6%) were benign tumours (seven Leydigomas and one capillary haemangioma) (Figs. 3), and 2/28 (7.1%) were non-neoplastic lesions (Leydig cell hyperplasia).

**Table 2:** Histology of the 39 operated lesions

Malignant tumours (*n* = 18)	Growing (*n* = 18)	Seminoma (*n* = 17) Non-seminoma (*n* = 1)
Benign tumours (*n* = 9)	Stable (*n* = 1)	Benign Leydigoma (*n* = 1)
	Growing (*n* = 8)	Benign Leydigoma (*n* = 7) Capillary haemangioma (*n* = 1)
Non-neoplastic lesions (*n* = 12)	Stable (*n* = 10)	Leydig cell hyperplasia (*n* = 6) Fibrosis (*n* = 3) Granulomatous orchitis (*n* = 1)
	Growing (*n* = 2)	Leydig cell hyperplasia (*n* = 2)

**Fig. 4:**
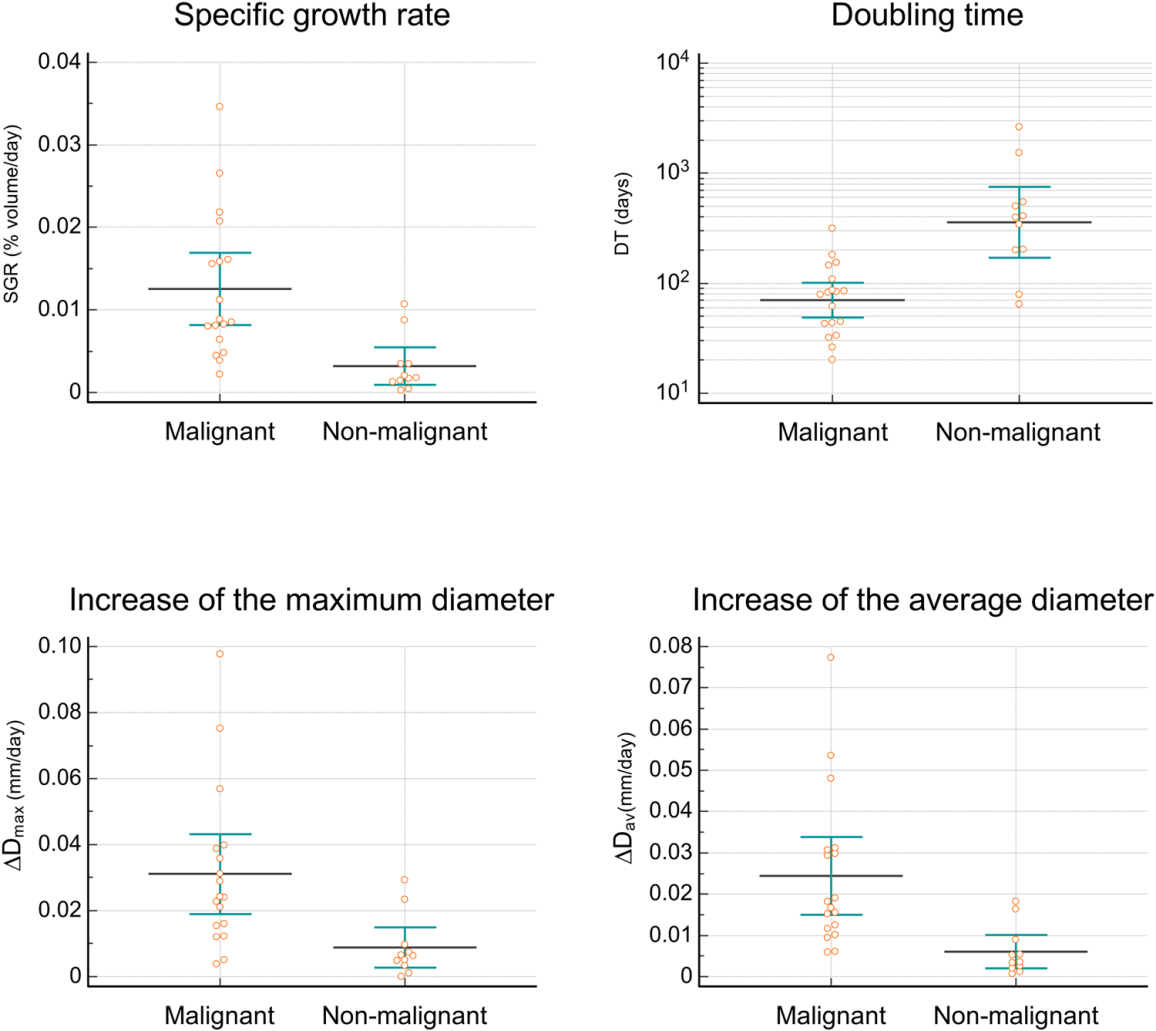
Specific growth rate (SGR, %/day), doubling time (DT, days), velocity of increase of the maximum diameter (∆D_max_, mm/day) and the average diameter (∆D_av_, mm/day) of malignant and non-malignant growing testicular incidentalomas

The initial volume of malignant and of non-malignant lesions was not statistically different (Student’s *t*-test, *p* = 0.53). Conversely, SGR, ∆D_max_ and ∆D_av_ were significantly higher for malignant tumours (11.4 × 10^−3^ vs 3.47 × 10^−3^%volume/day, 31 × 10^−3^ vs. 9 × 10^−3 ^mm/day, and 24 × 10^−3^ vs. 7 × 10^−3 ^mm/day, respectively, Fig. 4). There was a trend toward a higher DT for malignant tumours, but differences with non-malignant lesions were not statistically significant (Fig. 4, Table 3).

**Table 3:** Differences in growth indicators between surgically proved malignant vs. non-malignant lesions

Growth indicator	Malignant lesions^a^	Non-malignant lesions^a^	*p*
SGR (%volume/ day)	11.4 ± 2.11 × 10^−3^	3.47 ± 1.1 × 10^−3^	< 0.003
DT (days)	90 ± 17	535 ± 236	0.093
∆D_max_	31 ± 6 × 10^−3^	9 ± 3 × 10^−3^	< 0.003
∆D_av_	24 ± 4 × 10^−3^	7 ± 2 × 10^−3^	< 0.002

*SGR* specific growth rate (%volume/day), *DT* doubling time (days), *∆D*_*max*_ velocity of increase of the maximum diameter (mm/day), *∆D*_*av*_ velocity of increase of the average diameter (mm/day)

^a^ Data reported as average ± standard error of the mean

**Fig. 5:**
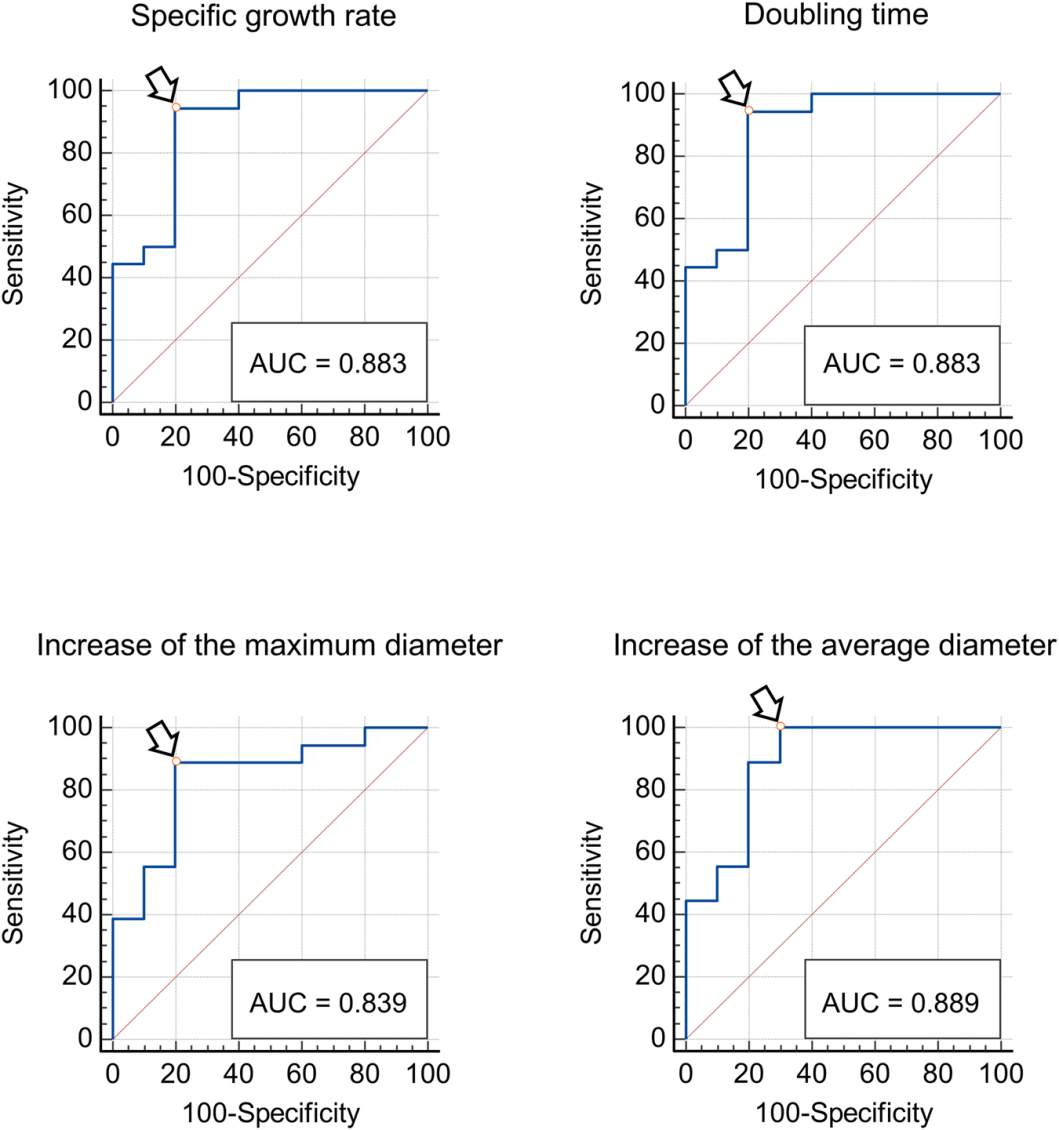
Receiver operating characteristic (ROC) curve analysis showing the diagnostic performance of specific growth rate (SGR, %/day), doubling time (DT, days), velocity of increase of the maximum diameter (∆D_max_, mm/day) and of the average diameter (∆D_av_, mm/day) for prediction of malignancy in patients with small (< 1 cm) growing testicular incidentalomas. The cut-off values of the growth indicators that maximise sensitivity and specificity (Youden index J) are indicated by the arrows

The best cut-off values of the growth indicators to differentiate between malignant and non-malignant histology were 3.47 × 10^−3^%volume/day, ≤ 179 days, > 10 × 10^−3 ^mm/day, and > 5 × 10^−3 ^mm/day for SGR, DT, ∆D_max_, ∆D_av_, respectively (ROC curve analysis, Youden index J) (Fig. 5, Table 4). All malignant tumours were removed within 18 months after discovery. They were all stage IA lesions.

**Table 4:** Area under the ROC curve (AUC) and cut-off values of the growth indicators that maximise both sensitivity and specificity to differentiate between surgically proved malignant vs. non-malignant testicular lesions

Growth indicator	AUC*	Associated criterion (Youden index J)	Sensitivity	Specificity
SGR (%/day)	0.883 ± 0.075	> 3.47 × 10^−3^	94.44	80.00
DT (days)	0.883 ± 0.075	≤ 179	94.44	80.00
∆D_max_	0.839 ± 0.082	> 10 × 10^−3^	88.89	80.00
∆D_av_	0.889 ± 0.071	> 5 × 10^−3^	100.00	70.00

*SGR* specific growth rate (%/day), *DT* doubling time (days), *∆Dmax* velocity of increase of the maximum diameter (mm/day), *∆Dav* velocity of increase of the average diameter (mm/day)

* AUC ± standard error

## Discussion

In this retrospective study, a consecutive series of 130 patients is investigated in whom small, non-palpable testicular tumours with negative tumour markers were initially followed up, with interval surgery undertaken only for growing lesions (*n* = 28), or patient/urologist preference (*n* = 11). Our data confirm that a conservative approach is effective in reducing unnecessary operations; surgery was avoided in 91/130 (70%) lesions.

For the 11 patients who were operated even though the lesion was stable during follow-up, all had benign tumours or non-neoplastic lesions. Conversely, 18/28 (64%) growing lesions were malignant, and evaluation of growth indicators helped identification of malignant tumours. The best cut-off values to differentiate between malignant and non-malignant histology were 3.47 × 10^−3^% volume/day, ≤ 179 days, > 10 × 10^−3 ^mm/day, and > 5 × 10^−3 ^mm/day for SGR, DT, ∆D_max_, ∆D_av_, respectively. An increase of the maximum diameter of the lesion of about one mm in three months and about two mm in six months, respectively, suggests malignancy and could lead to orchiectomy without further delay.

There is increasing evidence that orchidectomy constitutes overtreatment in patients with small incidentalomas [10], responsible for adverse fertility, hormonal, and mental health outcomes [11, 12]. Several investigations show that the majority of small testicular incidentalomas are benign or non-neoplastic lesions. Bieniek et al found six malignant lesions in 120 sub-centimetric testicular incidentalomas [3]. All malignant lesions were > 5 mm on initial imaging. Scandura et al investigated a series of 81 testicular incidentalomas < 1 cm and found that two-thirds of them, and all those < 5 mm were benign [10], with the conclusion that patients who undergo an orchidectomy for lesions < 5 mm are “Victims of Modern Imaging Technology”. In a recent systematic review, virtually all lesions < 3 mm and 86.6% of lesions < 5 mm were non-malignant [1]. Despite this, there remains a possibility that very small incidentalomas can represent small cancers. This is demonstrated in the current study, where the smallest malignant tumour identified had a volume of 176 mm^3^ and a maximum diameter of 4 mm.

Urologists are increasingly aware that a reasoned approach is necessary to reduce overtreatment while maintaining oncological safety. In these patients, radical orchidectomy should be avoided wherever possible, and in some cases, surgery may be avoided altogether. US surveillance is appropriate for the majority of testicular incidentalomas, where tumour markers are negative, and there are no clearly suspicious US characteristics [10].

Management strategies to reduce unnecessary surgery for small testicular incidentalomas include both improvement of lesion characterisation with multiparametric US [13–19] and magnetic resonance imaging (MRI) [20] and management based on the growth characteristics of the lesion at US. The Scrotal and Penile Imaging Working Group of the ESUR suggests active surveillance in these lesions, with US examinations every three months in the first year and then annually [9]. The World Federation of Ultrasound in Medicine and Biology recommends surgery for lesions that grow or display increasing vascularity on interval imaging, while stable lesions can be managed conservatively [17].

According to the ESUR recommendations, patients with small testicular incidentalomas should be monitored every three months for 12 months and then annually. How long lesions should be followed remains unclear. To the best of our knowledge, there is no generally accepted recommendation [17].

In principle, slow-growing, or stable seminomas cannot be excluded [21]. We therefore prefer to recommend follow-up of stable lesions indefinitely until a larger body of evidence is available to provide more robust guidance on the total duration of follow-up that is necessary.

In our series, 11 non-growing lesions were eventually operated due to patient and/or referring urologist preference. 10/11 of them were non-neoplastic lesions, one was a benign tumour. Similarly, in the series of Toren et al six non-growing lesions were operated; four were non-neoplastic lesions, 2 were benign tumours [4]. These data show that even though non-growing small incidentalomas are virtually all benign leaving a small testicular lesion in place presents a cause of anxiety for both urologists and patients and will result in overtreatment if surgery is performed. It is therefore desirable to provide reassurance and education to both urologists and patients about the safety of surveillance.

Although, in principle, malignancy cannot be excluded in non-operated incidentalomas this is unlikely.

Our data suggest that malignant small, non-palpable incidentalomas are mostly seminomas (17/18 in our series, 94%). All were identified and removed within 18 months after discovery, and all were stage IA tumours.

In clinical stage I seminoma tumour size has a bearing on the risk of tumour recurrence [22]. Therefore, it could be argued that a delay in the operation could be detrimental for the patient. For these small tumours, the increased risk of tumour recurrence is minimal. According to Chung et al, an increase in size from 1 cm to 2 cm raises the recurrence risk from 9% to 11% [23]. Therefore, the follow-up period should not adversely affect the patient’s prognosis.

In our series, only two patients had lesions > 2 cm in diameter when operated. They were a benign leydigoma, and a seminoma in a monorchid patient, who declined operation until the lesion reached a maximum diameter of 28 mm; this patient did not receive further treatment and is free from relapse after 10 years of surveillance.

We believe that based on these data and disease prevalence, a non-aggressive management of small testicular incidentalomas is justified, and should be considered safe when follow-up includes self-examination and tumour marker assessment, in order to reduce the risk of interval tumour growth [9].

Slow-growing seminomas can occasionally be encountered which cannot be distinguished from benign tumours based on growth characteristics [21]. In our series, a slow-growing seminoma was encountered (SGR 2 × 10^−3^%/day, DT 316 days). The lesion was stable during the first six months of follow-up and then began to increase in size. The patient initially resistant to surgery could have been eligible for testis-sparing surgery, but orchidectomy was performed.

In our series, SGR, ∆D_max_, and ∆D_av_, were significantly different for malignant and non-malignant lesions, while DT did not reach a statistical significance. The limitations of DT for quantifying tumour growth have been documented for pulmonary nodules [24].

The variability of DT is much larger than that for SGR, making the latter a more suitable measure of volume change. In particular, it has been shown that DT largely overestimates the difference in the growth rate of slowly growing tumours and underestimates the difference in the growth rate of rapidly growing tumours, while SGR uniformly indicates the difference between growth rates throughout all ranges [7, 25]. Consequently, the growth rate of testicular incidentalomas should be expressed by SGR, or percentage volume increase per unit time, when technically feasible. In our clinical practice, however, an overall increase in the greater diameter ≥ 1 mm during two consecutive follow-up investigations was considered an unequivocal growth. Serial evaluations are necessary due to the large intrinsic errors in the measurement of the diameters of sub-centimetric lesions.

For the same reason, SGR and DT are not routinely evaluated at the time of the examination, since errors in calculating the diameters are multiplied. This study has shown that linear parameters such as ∆D_max_, and ∆D_av_, which are immediately derived from the measurement of the diameters, are effective in providing a rough estimation of tumour growth, sensitive enough for prompt identification of fast-growing lesions. An increase in the maximum diameter of the lesion of about one mm in three months and about two mm in six months, in particular, suggests malignancy and could lead to orchiectomy without further delay. The use of high-end equipment and higher frequency probes improves spatial and contrast resolution. It could improve the accuracy of the measurements and make prompt identification of slow-growing lesions easier. Also, 3D imaging with automatic volume calculation could be an option to reduce variability.

This study has several limitations. The most important is the retrospective design and the relatively small number of operated patients. The timing of follow-up was not the same in all patients, as 29 patients had one or more missed examinations during follow-up. As is commonly accepted for small tumours, in this study exponential growth has been postulated for calculating SGR and DT [7], but this assumption requires confirmation with large prospective studies.

A second limitation regards the intrinsic inter- and intra-observer variability of measuring sub-centimetric nodules at US. Even though ultrasound technology continues to progress and the technical standards for measurements of distance have improved, a significant error cannot be excluded, when considering size differences of less than 1 mm. To reduce the impact of inaccurate measurement, one follow-up evaluation was not considered enough to affirm that the lesion was growing, but at least two consecutive measurements were deemed necessary, in which the greater diameter increased in both, and the overall increase was ≥ 1 mm. This means, for a nodule of 5 mm, an increase of the greatest diameter of 20%.

Another limitation of this study is that only one lesion was a non-seminomatous tumour, and only two non-neoplastic lesions were growing. This likely reflects the real prevalence of disease, suggesting that among small non-palpable incidentalomas with negative tumour markers that are growing, benign and malignant tumours prevail, malignancy is more frequent (64% in our series), and most malignant tumours are seminomas (94% in our series).

The very low incidence of non-seminomatous tumours in this series is difficult to explain since the overall prevalence of seminomas is approximately 55–60% [26]. However, about 85% of seminomas are diagnosed as clinical stage I disease as compared with about 60% among non-seminomas [27, 28]. This may be explained by a faster progression of non-seminomatous tumours [29]. Our findings may have a similar explanation. If non-seminomatous tumours grow faster, it may be less likely to identify them when they are non-palpable, < 1 cm lesions. Moreover, since about 58% of clinical stage I non-seminomatous tumours have positive tumour markers [27], some of these lesions could also have been excluded for this reason.

In conclusion, growing parameters allow an educated assessment of benign and malignant small testicular incidentalomas even though overlap exists. These results are promising, but the strength of the evidence suffers from the retrospective design of the study. Prospective multicentre follow-up studies using multiparametric US are necessary to substantiate our results. Further improvement in lesion characterisation is now offered by the increasing capabilities of multiparametric US. Changes of lesions characteristics in the different US modes during follow-up, considered together with growing parameters, are expected to further improve lesion characterisation, leading to an evidence-based paradigm shift in the management of small testicular incidentalomas.

## Abbreviations

DT: Doubling time
ESUR: European Society of Urogenital Radiology
ROC: Receiver operating characteristic
SGR: Specific growth rate
TSS: Testis-sparing surgery
